# Electroacupuncture and Acupuncture Promote the Rat's Transected Median Nerve Regeneration

**DOI:** 10.1155/2013/514610

**Published:** 2013-03-12

**Authors:** C. Y. Ho, C. H. Yao, W. C. Chen, W. C. Shen, D. T. Bau

**Affiliations:** ^1^School of Chinese Medicine, Institute of Chinese Medicine, China Medical University, 404 Yuh-Der Road, Taichung 40447, Taiwan; ^2^Departments of Family Medicine, China Medical University Hospital, 404 Yuh-Der Road, Taichung 40447, Taiwan; ^3^Department of Biomedical Imaging and Radiological Science, China Medical University Hospital, 404 Yuh-Der Road, Taichung 40447, Taiwan; ^4^Graduate Institute of Clinical Medical Science, China Medical University, 404 Yuh-Der Road, Taichung 40447, Taiwan

## Abstract

*Background*. Acupuncture and electroacupuncture treatments of damaged nerves may aid nerve regeneration related to hindlimb function, but the effects on the forelimb-related median nerve were not known. *Methods*. A gap was made in the median nerve of each rat by suturing the stumps into silicone rubber tubes. The influences of acupuncture and electroacupuncture treatments on transected median nerve regeneration were evaluated from morphological, electrophysiological, and functional angles. *Results*. Morphologically, the group receiving acupuncture and electroacupuncture treatments had larger total nerve area and blood vessel number compared with the controls. Electrophysiologically, the group receiving electroacupuncture had significantly larger amplitude and larger area of the evoked muscle action potentials compared with the controls. Functionally, the acupuncture and electroacupuncture treatments enhanced the injured paw's ability to regain its grasping power and resulted in a faster efficiency to a new bilateral balance. *Conclusion*. Our findings provide multiapproach evidence of the efficacy of acupuncture and electroacupuncture treatments to the regeneration of median nerve. Indeed, acupuncture and electroacupuncture appear to have positive effects on the regeneration processes. This platform is beneficial to further study the clinical application of acupuncture and electroacupuncture alternative treatments on nerve-injured patients.

## 1. Introduction

Peripheral nerve injury is an important issue in the world and represents a series of highly specialized processes of nerve regeneration [1]. The techniques of using silicone rubber to bridge a severed nerve provide a method for observing the regenerative processes. While most researches focus on the lower limbs injury model, upper limbs injuries are in fact more commonly observed in clinical trauma, especially median nerve injury [2, 3]. Recently, application of combining traditional Chinese acupuncture and electroacupuncture to stimulate nerve regeneration has become the mainstream treatment in clinical rehabilitation and related basic research [4, 5]. However, the effects of acupuncture and electroacupuncture were still controversial and seldom compared together, and had been evaluated from multiple approaches. Therefore, it is of great interest and urgent need to evaluate the influences of acupuncture and electroacupuncture on median nerve regeneration simultaneously and from multiple angles.

Acupuncture and electroacupuncture cause physiobiological effects that promote movement and function after peripheral nerve injury in individuals [6, 7]. Several researches have revealed that a low frequency electroacupuncture is a better approach to promote nerve regeneration after trauma injury [8, 9]. Therefore, it is reasonable to assume that electrical stimulation can have positive impacts on nerve regeneration. As a clinical physician, the clinical outcomes that resulted from rehabilitation medicine must be taken into consideration at first priority in which treatment is most likely to be safe and effective. Acupuncture and electroacupuncture provide a safe and effective way to help patients' rehabilitation after trauma accident. To the best of our knowledge, there are two pilot studies reported by Yao et al. using the conduit tube to investigate the effects of electrical stimulation at different frequencies and current levels on regenerating sciatica nerves [10, 11]. Besides Bertelli's model, there was little literature available that described the rat's upper limb's nerve injury model and discussed the different nerve regeneration rates, especially for common diseases like median nerve injury [12]. Because allograft and autograft nerve implantation lacks the availability of experimental models to evaluate the nerve regeneration condition, the nerve conduit provides a state-of-the-art platform. Therefore, the objective of this study is to determine whether acupuncture and/or electroacupuncture can serve as an effective clinical strategy for improving functional recovery after a median nerve transection.

## 2. Methods 

### 2.1. Silicone Rubber Tube Entubulation

Twenty-one adult Sprague-Dawley rats received placement of silicone rubber tube. First, all of the rats were surgical with inhalation anesthetic technique (AErrane; Baxter, Deerfield, IL, USA). The left arms and forelimbs of the rats were sheaved. Then, fascia and muscle were separated using blunt dissection after incising skin, and the left median nerve was severed into proximal and distal segments at, forelimb. Continuously the proximal and distal stumps were fixed with a simple 9-0 nylon direct suture through the nerve epineurium and silicone rubber tube (1.47 mm inter diameter, 1.96 mm outer diameter; Helix Medical, Carpinteria, CA, USA). Both the proximal and distal nerve segments were severed to the depth of 1 mm into the chamber, leaving a 5 mm gap between the bridge stumps. The muscle layer was sutured with 4-0 chromic gut sutures, and the skin was closed with 2-0 silk sutures. All animals were reared in temperature (22°C)- and humidity (45%)-controlled rooms with 12-hour light-dark cycles, and they had access to food and water ad libitum.

### 2.2. Acupuncture and Electroacupuncture Protocols

The acupuncture and electroacupuncture treatment protocols are similar to that previously reported [13]. Briefly, their left forearms were extended, and left paws were held in place by rubber tapes. One stainless steel needle electrode (0.35 mm outer diameter, 12 mm length) connected to the negative wick (cathode) of a stimulator (Trio 300, Ito, Japan) was inserted aseptically into the middle aspect of the wrist (Da-Ling, PC7), and another positive electrode (anode) was positioned around the site of the arm (Quze, PC3) along with pericardium meridian. The positive and negative stimulating sites were located near the proximal and distal ends of the implanted silicone tubes, respectively. The depth of insertion varied from 0.5 cm to 1 cm according to the thickness of skin and fatty tissues. The stimulation was applied to the animals for 15 minutes every other day beginning a week after the nerve repair. The reason that we did not perform the electrical stimulation on animals immediately after the nerve repair was to avoid the loosening of suture line on the skin because of muscle contraction, which might cause serious inflammatory reactions. All the animals were divided into three groups according to the current intensity of the electrical stimulation they received. In group A (*n* = 7), animals were the controls which received empty silicone rubber chambers, and the stimulator did not deliver current to the two stainless steel needle electrodes. In group B (*n* = 7), animals received a treatment of acupuncture stimulation after their injured nerves were bridged with the silicone rubber tubes. Similarly, animals in groups C (*n* = 7) received electrical stimulation of frequency 2 Hz, and the current intensity was 1 mA to produce a visual muscle contraction as the proper response of acupuncture.

### 2.3. Electrophysiological Measurements

After the entubulation duration of 5 weeks, all rats were reanesthetized, and the left hand median nerve was exposed. Then, the nerve was stimulated with supramaximal stimulus intensity through a pair of needle electrodes placed directly on the median nerve trunk, 5 mm proximal to the transection site. Latency, amplitude, muscle action potentials (MAPs) area, and nerve conduction velocity (NCV) were recorded from the thenar muscle with microneedle electrodes linked to a computer system (Biopac Systems, Goleta, CA, USA). The latency was recorded from the stimulus to the starting points of the first negative skew. The amplitude and the area under the MAP curve from the baseline to the maximal negative peak were measured. The MAP was used to evaluate the NCV, which was performed by placing the recording electrodes in the thenar muscle and stimulating the median nerve proximally. The NCV was then calculated by dividing the distance between the stimulating sites by the difference in latency period.

### 2.4. Histological Measurements

After the electrophysiologic MAP measurements, rapidly the regenerated median nerve was taken out. The median nerve sections were taken from the middle regions of the regenerated nerve in the chamber. After the fixation of glutaraldehyde (Merck, Whitehouse Station, NJ, USA), the nerve tissue was postfixed in 0.5% osmium tetroxide (Sigma Chemical Co., St. Louis, MO, USA), dehydrated in a series of graded alcohols (70, 80, 95, and 100%; Merck, Whitehouse Station, NJ, USA) for 60 minutes each, and embedded using a JB-4 Embedding Kit (Polysciences, Warrington, PA, USA). The tissue was then cut to 5 mm thickness by using a microtome with a dry glass knife and stained with toluidine blue (Sigma Chemical Co., St. Louis, MO, USA). Using an optical microscope (Olympus IX70, Olympus Optical Co., Japan) with an image analyzer system (Image-Pro Lite, Media Cybernetics, Bethesda, MD, USA), the number of neural components in each nerve section was counted. Myelinated axons in a frame of image randomly selected from each nerve specimen at a magnification of 400x were counted. When the axons in one image had been counted, those of a second image were counted, and so on until all images had been included. The total number of myelinated axons and their areas was then determined from the number of components in each image and the total number of images occupied by the nerve cross section. In addition, the total nerve areas including the epineurial and the endoneurial areas were measured under the microscope at 40x. Similarly, the areas of blood vessels were also measured.

### 2.5. Grasping Test Analyses

The analyzing system of a rats' grasp pattern by recording their grip power of the forepaw movements has been well established and widely employed for the assessment of motor nerve recovery after median nerve injury. In this study, a grasp methodology was designed by modifying that of Bertelli and Mira to assess the forelimb median nerve function of the rat [14]. Briefly, a mesh pull-bar assembly was placed via a threaded adaptor to a digital force gauge. The mesh was 8 cm × 15 cm inches with 2.5 square grids. The bars of the grids were 8 mm thick. The rats were gently lifted by holding their tails and then lowered toward the mesh and continued while the body was in a line where forepaw catch can reach for the mesh. When the digits of the forepaw had grasped the mesh, the rat was rapidly pulled vertically away from the mesh in a smooth and constant motion. The rats would hold onto the mesh until they can no longer resist the pull. When the grasp was broken, the report from the gauge was recorded as the grasp strength (Kgw, Newton). When the rats grasp the mesh, a digital video camera (Sony TCR19) was used to record their forelimb grasp movements. These movements were calculated three times, and the average of the measurements was taken. The measurements of the bilateral hands at 6 weeks postoperatively were recorded. All the measurements were done by the same observer and data expressed as mean ± SD.

### 2.6. Statistical Analysis

Statistical comparisons between groups were made by the one-way analysis of variance (SPSS 16.0). The Turkey test was then used as post hoc test.

## 3. Results 

**Figure 1:**
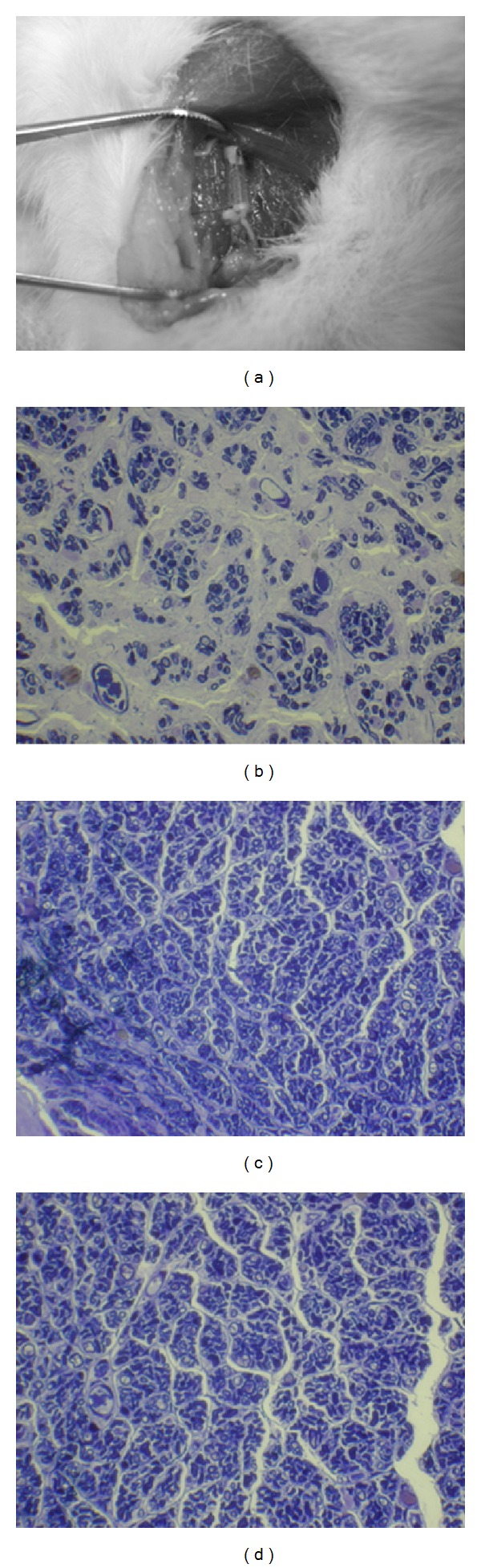
The sampling of median nerve from the rat (a) and light micrographs of the regenerated nerve cross sections of control (b); acupunctured (c), and electrical stimulation plus acupunctured (d) rats.

To focus on investigating the influence of acupuncture and electroacupuncture on the regenerative process, all other confounding factors, such as carious acupoints, permeability of biomaterial, surface morphology, and electrical properties, were eliminated by using nondegradable silicone rubber as the guide channel. After an implantation time of 5 weeks, the silicone conduit, together with the regenerated nerves in it, was then retrieved from the rats and evaluated. Swelling or deformation of the silicone tubes was not obvious, and no nerve dislocation was seen in all the investigated rats. It was found that the regenerated nerve, which was surrounded by fluid, occupied a central location within the tube (Figure 1(a)). Rough examination of the silicone rubber chambers revealed 100% rates of successful nerve regeneration in all the three groups (A: control, B: acupuncture, and C: electroacupuncture groups) with the animals exhibiting a regenerated nerve cable across the 5 mm gap. Figures 1(b)–1(d) showed representative cross sections of nerve specimen retrieved from the A (Figure 1(b)), B (Figure 1(c)), and C (Figure 1(d)) groups. The influence of acupuncture and electroacupuncture on the regeneration of the nerve will be investigated from three angles: nerve morphology, nerve electrophysiology, and integrate function of nerve-recovered grasping capacity.

**Figure 2:**
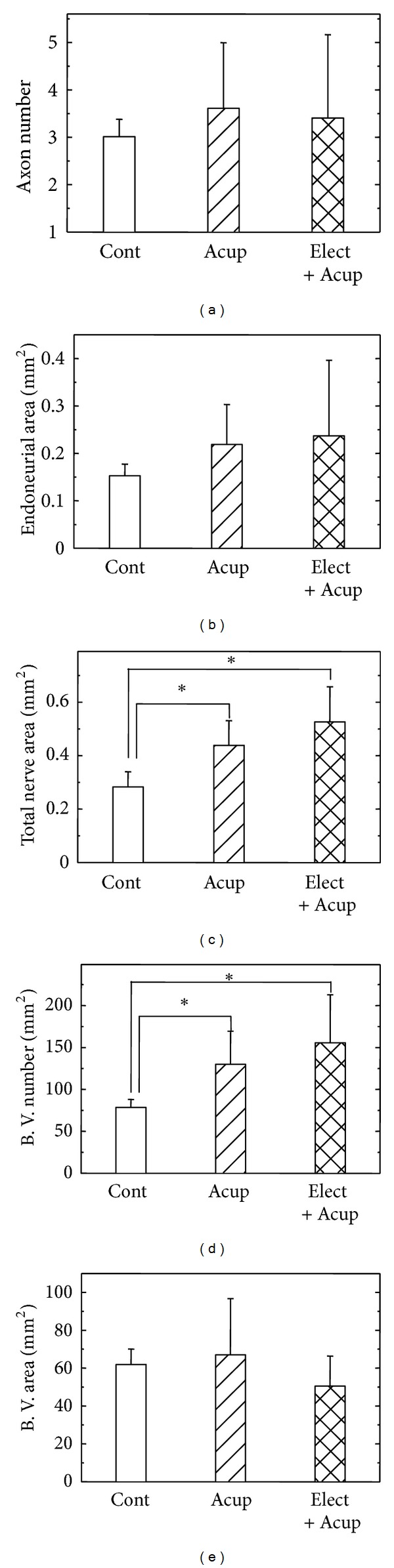
Morphometric analysis from the regenerated nerves in the chambers receiving electrical stimulation at different current intensities, including axon number (a), endoneurial area (b), total nerve area (c), blood vessel number (d), and blood vessel area (e). Cont: untreated control group; Acup: acupuncture group; Elect + Acup: electroacupuncture group. *Statistically different compared with the control group, *P* < 0.05.

First, the morphometry examined with quantification helped us to understand the alterations of nerve regeneration and the effects of acupuncture and electroacupuncture on nerve regeneration (Figure 2). The indexes included the mean values of axon number, endoneurial area, total area, blood vessel number, and blood vessel area, which were positively indexes for better regeneration. In the results, we can find that in the acupuncture and electroacupuncture groups, the total nerve areas and blood vessel numbers were larger than those in the control group (*P* < 0.05). Also, all the other indexes in the acupuncture and electroacupuncture groups seemed to be slightly higher than those in the controls, except the areas of blood vessel in the electroacupuncture group (Figure 2 and Table 1).

**Figure 3:**
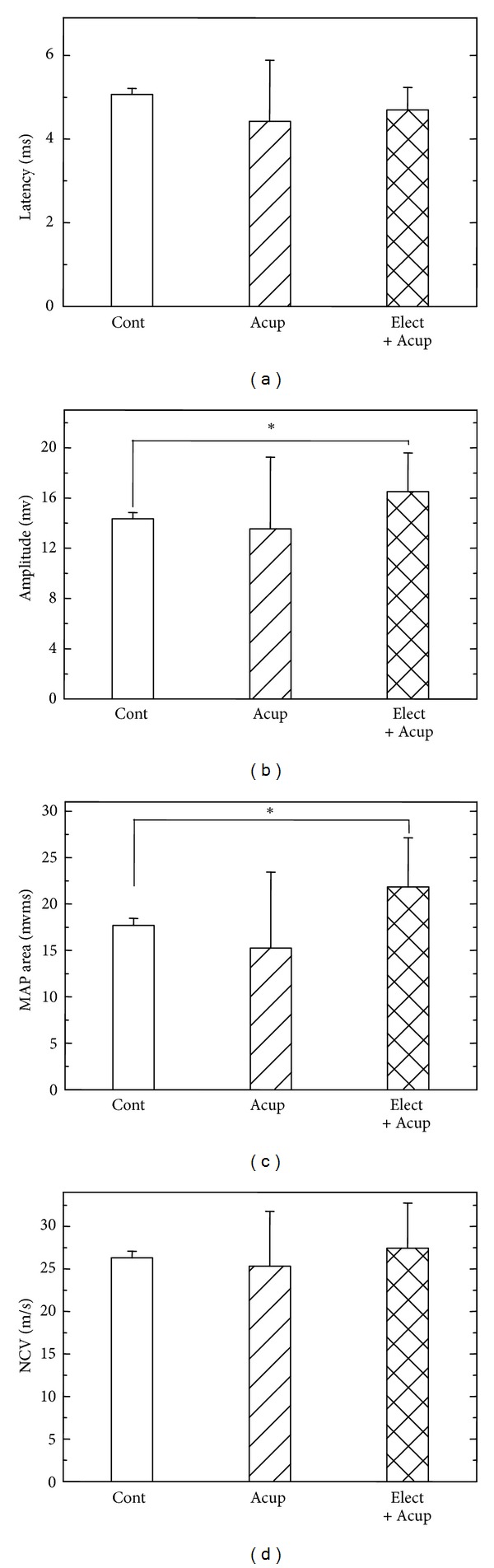
Electrophysiological analysis of the evoked MAPs, including latency (a), amplitude (b), area under the MAP curves (c), and NCV (d). Cont: untreated control group; Acup: acupuncture group; Elect + Acup: electroacupuncture group. *Statistically different compared with the control group, *P* < 0.05.

Second, we were interested in the effects of acupuncture and electroacupuncture on the reconstruction of nerve virtue electrofunction. To estimate the effect from multiple approaches, the MAP indexes included the latency, the amplitude, the MAP area, and the NCV as described in Section 2. As for the MAP indexes, the regenerated nerves treated with electrical stimulation in addition to acupuncture had relatively larger amplitude and larger MAP area compared with the controls (Figure 3). The latency was slightly shorter, and the NCV was slightly faster. As for the acupuncture group, the effects were too small to make any difference in the electrophysiological measurements (Figure 3 and Table 1).

**Figure 4:**
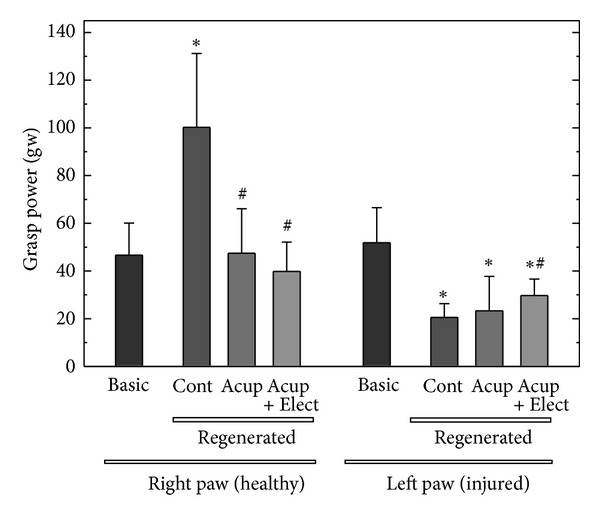
Functional analysis of the grasping power from the right (healthy) and left (injured) paws of the rats. Cont: untreated control group; Acup: acupuncture group; Elect + Acup: electroacupuncture group. *Statistically different compared with the basic (intact) group, *P* < 0.05; ^#^statistically different compared with the control group, *P* < 0.05.

Finally, we investigated the integrate function of nerve regeneration by analyzing the recovered grasping capacity (Figure 4). In the design, we had measured not only the grasping capacity of the injured left paw but also the healthy right paws as well. The results showed that in the control group, the injured paw had regained only 39.6% of the original strength after nerve regeneration. In the other two groups, acupuncture and electroacupuncture improved the recovery rates up to 45% and 57.4%, respectively. Obviously, the electroacupuncture treatment had a better and significant improvement compared with the controls while grasping the mesh (*P* < 0.05).

## 4. Discussion

It is believed that acupuncture and electroacupuncture would do well to ameliorate nerve regeneration and movement function recovery. However, there is very few evidence available to support this theory. In the previous literature, it has been reported that low frequency electroacupuncture and acupuncture could improve sciatica nerve regeneration [10, 12, 15–17]. Koppes et al. indicated that alternation of neurite growth could be manipulated by extracellular direct current [18]. The in vitro nerve growth promoting effects of electrical stimulation have also been demonstrated with the in vivo experiments showing that nerve regeneration could be enhanced by applying direct current to the sciatic nerve of rats as the cathode was placed toward the distal end of the injured nerve conduits. Zhang et al. provided the peripheral nerve regeneration with a definite theory stating that the nerve's successful rate was decided via the balance of contact guidance substance, basement microtube formation, neurotrophic factor, and contractile fibroblast capsule [19, 20]. McCaig et al. revealed that electroacupuncture could stimulate two cascade pathways: one was related to the activation of phospho-inositide-3 kinase (PI-3K) and phospholipase C (PLC); the other is to trigger laminin to secret integrins [21]. At the same time, the electroacupuncture could lead to an increase of calcium concentration and consequently an increase in the concentration of cAMP and cAMP-dependent protein kinase A, which would promote nerve regeneration [21]. It has also been explored that placing direct current at the cathode would form adhesion-associated proteoglycan to accelerate nerve regeneration [22]. In our previous researches in which we studied injured nerves related to the hindlimbs, we recognized that acupuncture and electroacupuncture could accelerate the maturity of regenerated nerves with larger mean values of axon number, endoneurial area, blood vessel number, and blood vessel area as compared with the controls [13, 23]. This was similar to our current study of the injured median nerve related to the forelimbs. The electroacupuncture could significantly improve the overall recovery at the indexes of the enlarged total nerve area, blood vessel numbers, nerve amplitude, and MAP area. Most importantly, electroacupuncture could help our rats to regain grasping power (Figures 2–4 and Table 1). However, in our current study, the effects of acupuncture were only significant in the increasing of the total nerve area and blood vessel numbers.

Chen et al.'s and Lu et al.'s studies revealed that acupuncture and electroacupuncture increase regenerated nerve Schwann cell proliferation and provide blood supply for treatments. Some investigators had pointed out that when the nerve was transacted, the electrical stimulation would help blood reconstruction via somatic/autonomic reflex arc to provide enough blood flow for the regenerated nerve [11, 13]. In our study, the effects of acupuncture and electroacupuncture on blood vessel reconstruction were not as obvious as the previous findings, which may also be due to the different targets that were investigated. In his experiment, Bertelli and Mira first used the median nerve model and grasp test for the evaluation of functional median nerve recovery [12, 14]. As a result, the regenerated nerves treated with electroacupuncture had relatively shorter latency, larger amplitude, and larger MAP area as compared with the controls. These results indicated that the transected nerves receiving acupuncture and electroacupuncture have undergone an enhanced regeneration with more mature nerve fibers that have reinnervated the muscle fibers [12, 14]. Although it is uncertain whether electrical stimulation promotes an outgrowth of neural components in developing nerves, the movement function-improving capability of electrical stimulation on regenerated nerves is obvious. The kinematic grasp analysis to median nerve function evaluation is usually designed to assess individual upper motor functions, which can prevent the interference of compensatory movements from healthy limbs. However, in previous designs, the healthy limb's function was not measured simultaneously as ours. It is interesting to find that the rats in the control group depended so much on the healthy right paw that the grasping strength could reach almost twice as much as the other groups after the duration of the nerve regeneration (Figure 4). More interestingly, the rats in the acupuncture and electroacupuncture groups exhibited better balance and coordination between their left and right limbs. In other words, the acupuncture and the electroacupuncture treatment would do more benefit to enhance the injured forepaw grasp strength but reduce the dependency on the healthy forelimb than the control groups. It is promising that median nerve injured patients receiving acupuncture and electroacupuncture treatment may recover faster and better and may regain a bilateral balance of normal life. 

This study provides a better strategy to assist the recovery of nerve-injured patients in clinical practices. However, there is still much work to be done before this practice can be performed in a real setting. These include an overall examination of the various types of electrical stimulation (continuous or pulsed), meridian acupoints, the stimulation parameters, the sites for the placement of electrodes, and most importantly the length of the nerve gap, which may affect the efficacy of electrical stimulation on nerve regeneration. Our animal models provide a platform for further studies on establishing a palette of electrical stimulation with different stimulus combinations and an efficient multiapproach examination for figuring out the optimal way to promote the growth of the regenerating nerves.

## 5. Conclusions

The current study provides evidence indicating that traditional acupuncture and electroacupuncture have potential rehabilitating effects on the regeneration of the dissected median nerves from the angles of morphology, electrophysiology, and grasping function.

## Figures and Tables

**Table 1:** The morphometric, electrophysiological, and grasping analysis of the influences of acupuncture and electroacupuncture on nerve regeneration.

Measuring index	Control	Acupuncture	Electroacupuncture
Morphometric analysis			
Axon number^#^	3009 ± 977	3612 ± 1381	3404 ± 1528
Endoneurial area (mm^2^)	0.15 ± 0.06	0.22 ± 0.09	0.24 ± 0.15
Total nerve area (mm^2^)	0.28 ± 0.15	0.40 ± 0.17*	0.52 ± 0.17*
B. V. number^#^	78 ± 25	130 ± 39*	154 ± 63*
B. V. area (*μ*m^2^)	62 ± 21	67 ± 30	51 ± 17
Electrophysiological analysis			
Latency (ms)	5.11 ± 0.8	4.39 ± 1.02	4.69 ± 0.57
Amplitude (mv)	12.49 ± 1.31	12.82 ± 3.67	16.50 ± 3.18*
MAP area (mvms)	16.41 ± 2.96	15.54 ± 5.57	21.85 ± 5.17*
NCV (m/s)	24.07 ± 1.3	24.97 ± 4.55	27.44 ± 5.58
Grasping analysis			
Right paw (healthy)	100.26 ± 30.94	47.45 ± 18.66*	39.80 ± 12.33*
Left paw (injured)	20.51 ± 5.80	23.31 ± 14.44	29.74 ± 6.86*

*P <0.05 compared with the control group; B. V.: blood vessel; MAP: muscle action potential; NCV: nerve conductive velocity.

^#^Cell number.
